# ASO Author Reflections: The Beneficial Effect of High-Volume Center Experience on Surgical Outcomes After Total Pancreatectomy

**DOI:** 10.1245/s10434-020-08986-6

**Published:** 2020-08-11

**Authors:** Thomas F. Stoop, Marco Del Chiaro

**Affiliations:** 1 Pancreatic Surgery Unit, Division of Surgery, Department of Science Intervention and Technology (CLINTEC), Karolinska Institutet at Department of Upper Abdominal Diseases, Karolinska University Hospital, Stockholm, Sweden; 2 Department of Surgery, Cancer Center Amsterdam, Amsterdam UMC, University of Amsterdam, Amsterdam, The Netherlands; 3 Division of Surgical Oncology, Department of Surgery, University of Colorado, Anschutz Medical Campus, Aurora, CO USA

## Past

The role of total pancreatectomy (TP) is historically highly debatable. TP has failed to show a more radical outcome in the treatment of pancreatic cancer in comparison with partial pancreatectomy.^1^ Moreover, TP has been associated with severe metabolic sequels and potentially life-threatening long-term complications;^2^ however, for some indications, TP remains the only treatment option for some pancreatic diseases.^3^

## Present

In recent years, the number of TPs worldwide has increased, since its subsequent endocrine and exocrine insufficiency seems to be better manageable, with an acceptable impact on quality of life.^4^ Some ‘new’ pancreatic diseases (i.e. intraductal papillary mucinous neoplasms [IPMNs]) require a TP in some cases in order to achieve curation.^5^ At the same time, with the improvement in the systemic treatment of pancreatic cancer,^6^ TP can be used in cases of locally extended disease.

In the Karolinska University Hospital, the annual relative and absolute number of TPs has also increased,^7^ mainly as a consequence of a lower threshold for performing TPs for IPMNs and adenocarcinomas, particularly through intraoperative decision making to carefully select only those patients in which TP will be of oncological benefit. Surgical outcomes improved significantly as surgical volumes increased to more than 20 TPs per year, especially after TP with concomitant resection of adjacent structures.

The current satisfying results of TP without extended resections justify its role as treatment for diffuse or multifocal premalignant pancreatic diseases. Besides the oncological benefit of TP as a treatment option for resectable pancreatic cancer with repetitive isolated neck margin, the value of extended TP for borderline resectable and locally advanced pancreatic cancer seems to be feasible and safe if performed in high-volume, experienced centers.

## Future

The present study implicates that TP should be preferably performed in high-volume, experienced centers, especially in cases of extended resections; however, larger series are required to precisely investigate the short- and long-term surgical and oncological outcomes after both standard and extended TP compared with partial pancreatectomies. These results also need to be carefully weighted with the long-term consequences in terms of quality of life and chronic complications.
